# Low value for the static background dielectric constant in epitaxial PZT thin films

**DOI:** 10.1038/s41598-019-51312-8

**Published:** 2019-10-11

**Authors:** Georgia Andra Boni, Cristina Florentina Chirila, Luminita Hrib, Raluca Negrea, Lucian Dragos Filip, Ioana Pintilie, Lucian Pintilie

**Affiliations:** National Institute of Materials Physics, Atomistilor 405A, Magurele, Ilfov Romania

**Keywords:** Ferroelectrics and multiferroics, Ferroelectrics and multiferroics

## Abstract

Ferroelectrics are intensively studied materials due to their unique properties with high potential for applications. Despite all efforts devoted to obtain the values of ferroelectric material constants, the problem of the magnitude of static dielectric constant remains unsolved. In this article it is shown that the value of the static dielectric constant at zero electric field and with negligible contribution from the ferroelectric polarization (also called static background dielectric constant, or just background dielectric constant) can be very low (between 10 and 15), possibly converging towards the value in the optical domain. It is also found that the natural state of an ideal, mono-domain, epitaxial ferroelectric is that of full depletion with constant capacitance at voltages outside the switching domain. The findings are based on experimental results obtained from a new custom method designed to measure the capacitance-voltage characteristic in static conditions, as well from Rayleigh analysis. These results have important implications in future analysis of conduction mechanisms in ferroelectrics and theoretical modeling of ferroelectric-based devices.

## Introduction

Although ferroelectric materials have been studied for the better part of the last 100 years, the magnitude of the static dielectric constant of these materials is still a matter of debate and controversy. For a long time, it was thought that ferroelectrics are materials with large values of the static dielectric constant^1^. However, this is valid for ceramics or polycrystalline films, while in the case of epitaxial layers it was found that the dielectric constant is significantly smaller^2–4^. Even when considering the same material, e.g. BaTiO_3_ (BTO), the reported values for the dielectric constant span an interval of at least one order of magnitude, from about 100 to more than 1000, generating difficulties in developing theoretical models involving this quantity^5–7^. Therefore, it is fair to consider that the static dielectric constant in ferroelectric materials is very much sample dependent, being affected by extrinsic contributions coming from various structural defects that may act as trapping centers for electric charges that may be able to respond to the small amplitude *ac* signal used for standard capacitance measurements^8^. It immediately follows that, if one intends to get closer to the intrinsic value of the dielectric constant in ferroelectrics, it has to prepare single crystal or epitaxial samples with reduced density of structural defects^9^. Values well below 100 can be obtained in epitaxial films of reduced thickness^4,9,10^, and as low as 17 in a 12 nm thick (Ba,Sr)TiO_3_ thin film^11^.

The reduction of the dielectric constant with the thickness of the epitaxial ferroelectric layers was attributed to the presence of so called dead-layers at the electrode interfaces, namely layers that are not ferroelectric and, therefore, have low value of dielectric constant^12–15^. The debate on whether dead-layers are present at the electrode interfaces is closely related to the debate regarding the partial or total depletion of the ferroelectric capacitors, and it is largely accepted that ferroelectric-metal interfaces behave as Schottky contacts^16–20^. Therefore, many experimental and theoretical studies were devoted to these problems for the past 20 years, some considering only dead-layers at the interfaces, some showing that there are no such things as dead-layers, some considering only depletion regions, and some considering both depletion and dead-layers^1,21–33^.

Returning to the subject of this study, it is known that the static dielectric constant can be estimated from the results of capacitance measurements, such as capacitance-voltage (C-V) characteristics, also the presence of ferroelectricity can be assessed if well-known butterfly shape is obtained. Standard C-V measurements involve simultaneous applications of two voltages on the sample: a *dc* voltage used to set the polarization value and direction, and a small amplitude (relative to the coercive voltage) *ac* voltage for determining the capacitive response measured with a standard LRC bridge. The C-V measurement described above is a type of dynamic measurement, as the *dc* voltage is varied step by step, such that the *dc* voltage is set to a certain value and the capacitance is measured using the small amplitude *ac* voltage. After recording the capacitance value, the *dc* voltage is set to the next value and the procedure is repeated until the full C-V characteristic is obtained^34–37^. The problem that arises is the parasitic contributions appearing in the measured capacitance due to transitory phenomena induced by the step change of the *dc* voltage (e.g. charge carriers released from traps able to respond to the frequency and amplitude of the *ac* voltage)^38^. All these issues will be reflected in the value of the calculated dielectric constant!

It is fair to assume that, in a ferroelectric capacitor, the dielectric constant can be divided in two parts: one related to ferroelectric polarization and one related to the dielectric response when there is no contribution from ferroelectric polarization (the case of complete saturation). The static dielectric constant, when there is no contribution from ferroelectric polarization, is sometimes called background static dielectric constant and should be related to the linear dielectric response associated to any non-ferroelectric material^39,40^.

The background static dielectric constant can be introduced with the help of the following equation:

1 $$D={\varepsilon }_{0}E+P$$

here, *D* is the electric displacement, *E* is the electric field, *ε*_0_ is the vacuum permittivity, and *P* is the total polarization, including the linear part *P*_*L*_ specific for any dielectric/semiconductor material, and the non-linear ferroelectric part characterized by the spontaneous polarization *P*_*S*_. Replacing *P*_*L*_ with *ε*_0_*χE* (*χ* is the electric susceptibility), Eq. (1) can be written as:

2 $$D={\varepsilon }_{0}(1+\chi )E+{P}_{S}={\varepsilon }_{0}{\varepsilon }_{b}E+{P}_{S}$$

here *ε*_*b*_ is the static background dielectric constant. The total static dielectric constant of a ferroelectric *ε*_*f*_ can be defined as:

3 $${\varepsilon }_{f}={\varepsilon }_{b}+\frac{1}{{\varepsilon }_{0}}\frac{\partial {P}_{S}}{\partial E}$$

It is clear from Eq. (3) that, if the spontaneous polarization is saturated and no longer varies with the applied electric field, then *ε*_*f*_ reduces to *ε*_*b*_.

The question is: how large is the static background dielectric constant? In this paper we suggest that the static background dielectric constant of an epitaxial ferroelectric layer can have values as low as 10–15 and may converge towards the optical dielectric constant, even in the low frequency domain, in defect free, very thin epitaxial films. In order to sustain this claim, a new C-V measurement procedure, named “static” C-V, was developed to estimate the values of the dielectric constant in conditions as close as possible to the electrostatic ones. The differences compared to the results of standard C-V measurements are discussed in terms of Schottky contacts that are present at the electrode interfaces. Rayleigh analysis was also performed to confirm the results and, by comparing them with those extracted from static C-V, it was possible to estimate the polarization contribution at small amplitude ac electric fields used for capacitance measurements.

## Results

**Figure 1:**
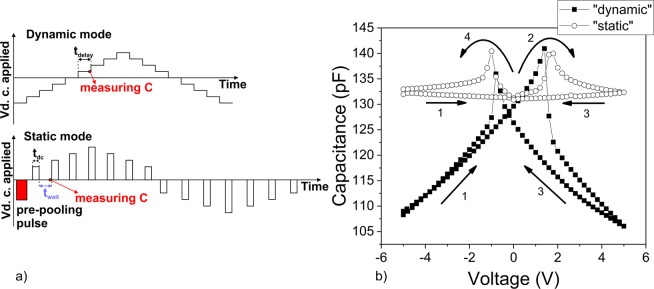
(**a**) The voltage pulse sequence for the “dynamic” and “static” C-V: *t*_*delay*_ is the delay time between changing the *dc* voltage and measuring the capacitance in the “dynamic” mode; *t*_*dc*_ is the time for applying the *dc* voltage that sets the polarization state in the “static” mode; *t*_*w*_ is the waiting time after removing the *dc* voltage; (**b**) the “static” and “dynamic” C-V characteristics for the 150 nm thick sample (pre-poling pulse of 0.1s; *t*_*dc*_ = 0.1s; *t*_*w*_ = 1s; frequency of the *ac* voltage of 100 kHz).

The results were obtained on a set of epitaxial Pb(Zr_0.2_Ti_0.8_)O_3_ (PZT) films having thicknesses of 20, 50 and 150 nm. The proposed “static” C-V measurement uses the following procedure: a pre-poling pulse of a certain period is applied on the sample in order to set the polarization in the –*P* state; the pre-poling voltage is removed and the capacitance is measured with the small amplitude *ac* signal after a waiting time (this is the first point, corresponding to 0 V, thus to the remnant polarization; the waiting time is necessary to allow relaxation of transitory phenomena); the *dc* voltage is set to a positive value slightly higher than zero and it is applied on the capacitor for a certain period of time; the *dc* voltage is removed and the capacitance is measured again, after the waiting time, at zero volt but with polarization set by the positive voltage previously applied on the sample; the *dc* voltage is set to a positive value slightly higher than the previous one and applied to the sample for the same period of time as the previous one; the *dc* voltage is removed again and the capacitance is measured after the same waiting time; the procedure is repeated step by step until a full hysteresis cycle is obtained (see the *dc* voltage sequence in Fig. 1a). One can observe that, contrary to the standard dynamic C-V, in this case no *dc* bias is applied on the sample while the capacitance is measured with the small amplitude *ac* signal. The resulting C-V characteristic is presented in Fig. 1b) together with the one obtained after a standard C-V measurement using staircase like increase of the *dc* voltage. The waiting time was set to 1 second after checking that it is long enough to allow relaxation of all transitory phenomena (see Supplementary Information SI).

**Figure 2:**
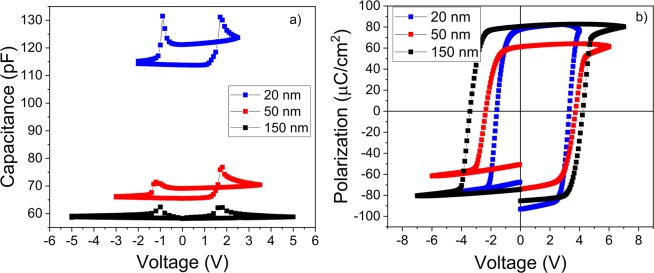
(**a**) The “static” C-V characteristics for epitaxial PZT layers of different thicknesses (the measuring times are the same as in Fig. 1b; the frequency of the ac voltage was 100 kHz); (**b**) the hysteresis loops recorded for epitaxial PZT films of different thicknesses (triangular voltage with 1 kHz frequency).

It can be seen that the C-V characteristic obtained using “static” C-V measurement retains the butterfly shape but, contrary to the standard C-V measurement (which from now on will be referred to as “dynamic”), the capacitance value has a very small variation with the *dc* voltage after achieving the saturation of the ferroelectric polarization. In fact, when the *dc* voltage is swept down from maximum value to zero, the capacitance variation is below 1%, being virtually voltage independent as it should be if the polarization is saturated. Similar results were obtained on samples with other thicknesses (see Fig. 2a) and for different frequencies (see details in SI). One can see that the capacitance remains constant after switching, once the polarization is saturated.

An interesting observation is that, for the samples of 20 and 50 nm thickness, the “static” capacitance has different values for sweeping the voltage up and down between maximum negative and positive voltage values. This may be related to the presence of an internal electric field, as evidenced from the hysteresis loops presented in Fig. 2b). As the thickness increases, the intensity of the internal electric field reduces, the hysteresis loop becomes more symmetric and the differences in the capacitance values obtained when the *dc* voltage is swept up and down becomes negligible as it is the case for the 150 nm thick sample.

**Figure 3:**
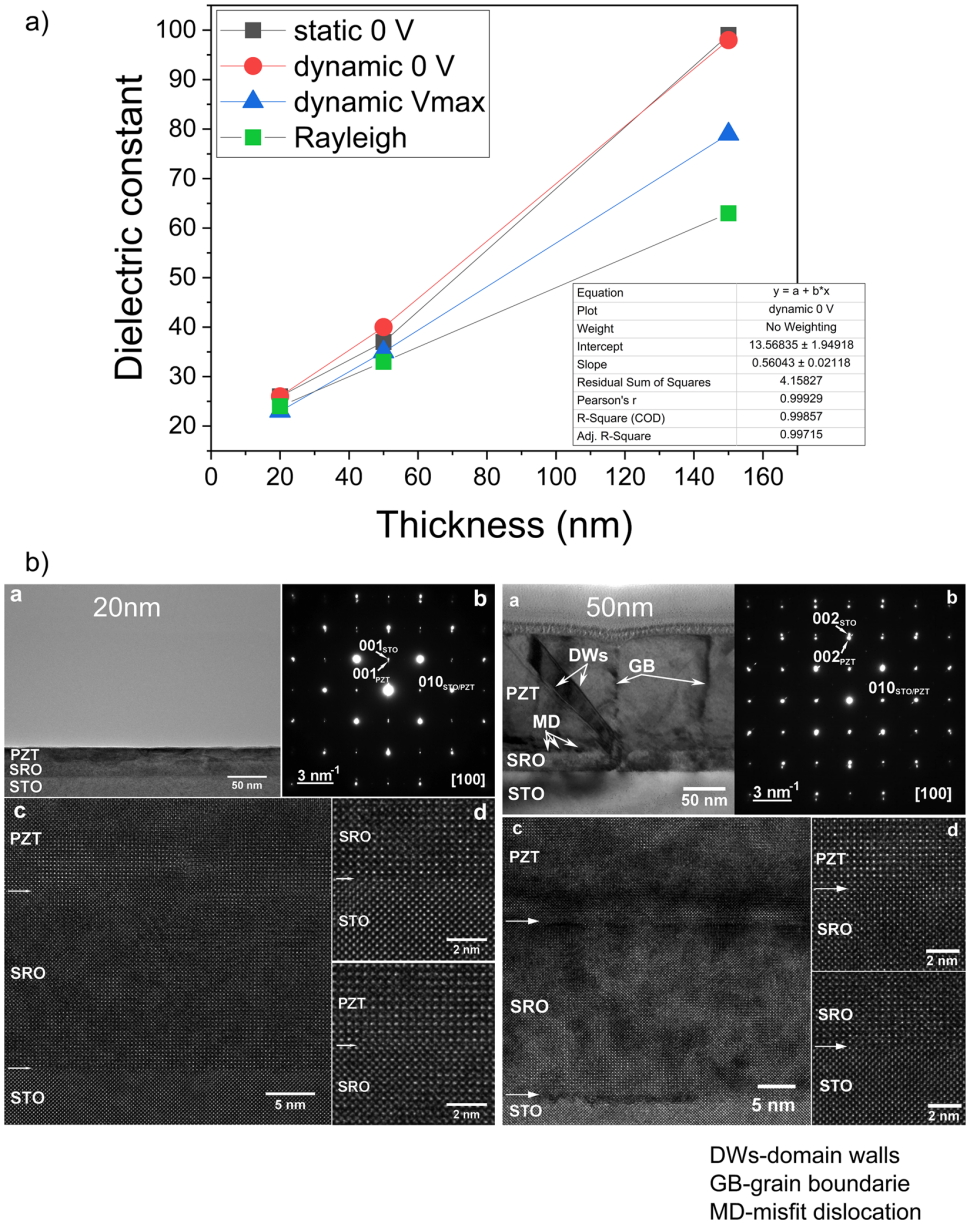
(**a**) The thickness dependence of the dielectric constant evaluated from C-V measurements performed at 100 kHz. Evaluation was performed in three cases: at 0 V “static”; at 0 V “dynamic”; at maximum applied voltage “dynamic”. (**b**) TEM images for 20 nm and 150 nm thick samples (inside each image the notations are a-low magnification image cross-section; b-SAED image; c-low magnification HR-TEM image; d-high magnification HR-TEM image of PZT/SRO interface and SRO/STO interfaces; these images demonstrate the high quality of the epitaxial growth).

The dielectric constant was estimated from capacitance values for several situations: at 0 V-“static” measurement (average between capacitances measured while sweeping up and down the dc voltage); at 0 V-“dynamic” measurement (average between capacitances measured while sweeping up and down the dc voltage); at maximum applied V-“dynamic” measurement (average between capacitances corresponding to maximum negative and positive *dc* voltages). The results are presented in Fig. 3a) as function of sample thickness.

Considering that the hysteresis loops are almost rectangular (see Fig. 2b), one can assume that the ferroelectric polarization is saturated when the voltage goes down to zero from the maximum applied *dc* voltage. The amplitude of the *ac* voltage used for capacitance measurements is of 100 mV, at least one order of magnitude smaller than the coercive voltage. One can be tempted to assume that the polarization contribution is negligible in the case of the three situations mentioned above, and that the estimated value is the static background dielectric constant *ε*_*b*_. However, one cannot exclude small, reversible, polarization variations at electric fields below the coercive one (usually below half of the coercive field, as it is the case for the amplitude of the *ac* voltage used for capacitance measurements). These may contribute to the static dielectric constant even the *dc* bias is removed, as it is the case for static C-V. Therefore, Rayleigh analysis was performed on the three samples, following the procedure described in refs^41,42^ (see details in SI). The values estimated for the dielectric constant at zero field, using the Rayleigh analysis, are also presented in Fig. 3a).

One can observe from Fig. 3a) that the values estimated from the C-V characteristics at zero volt are about the same, while the values at maximum applied *dc* voltage during dynamic measurements are significantly lower, especially as the thickness increases. Also, one can observe that all the methods give about the same value for the thinnest sample, around 23–25, while for the thicker samples the values obtained from C-V measurements are slightly higher than those obtained from Rayleigh analysis. One may infer that the difference is due to the response of the ferroelectric polarization to the small ac voltage used for capacitance measurements. The contribution of the ferroelectric polarization is increasing with thickness as 90^0^ domains start to develop in the sample of 150 nm thickness, as shown in TEM investigations presented in Fig. 3b). Based on these results, one may assume that the values obtained from Rayleigh analysis are closest to the static background dielectric constant, and that in very thin epitaxial films the polarization contribution to the static dielectric constant estimated from C-V measurements can be well below 10% (see SI). These results will be further discussed in the next section.

## Discussion

Figure 3a) suggests a linear dependence of the dielectric constant on sample thickness (see the inset, showing that the linear fitting has a confidence factor of 0.99). Assuming that this empirical dependence continues to remain true for lower thicknesses, then a value of about 14–15 is obtained for the background dielectric constant in samples around 1.2 nm in thickness, which are still ferroelectric according to literature^43–46^. This value is about twice the value of 6–7 reported to date for the optical dielectric constant of PZT type materials^47^.

Therefore, the value of *ε*_*b*_ can be as low as 14–15 in ultra-thin epitaxial layers, possibly including a minimal contribution from polarization (well below 10%) if a small amplitude *ac* voltage is used to measure the capacitance. A value virtually free of polarization contribution may be obtained using Rayleigh analysis, but this is hard to apply to very thin films due to larger leakage compared to thicker films. Another contribution comes from the structural defects that may be charged and may still respond to the low amplitude *ac* signal used for capacitance measurements in the low frequency domain. Such defects, especially point defects (e.g. vacancies), are present even in high quality, very thin epitaxial films. More defects (e.g. dislocations) can form as the thickness increases and the film start to relax from the strain imposed by the substrate, as can be seen in TEM images presented in Fig. 3b). This can also contribute to the larger static dielectric constant in thicker films. In a perfect epitaxial film, the contribution from structural defects vanishes and the value of the dielectric constant can be lower. One can imagine the extreme case of an ideal mono-domain ferroelectric film where the static background dielectric constant is only slightly larger than the optical dielectric constant, due to a very small contribution of the ferroelectric polarization. This result, based on experimental findings, confirms the early predictions of Watanabe *et al*. that the value of background permittivity in ferroelectrics may be close to the vacuum permittivity^48^.

Theoretical calculations performed on bulk crystal, as well as early experimental findings based on Raman measurements performed on bulk samples, report values of the dielectric constant of about 80 (thermodynamic theory applied to PZT with Zr/Ti ratio of 20/80)^49^, or around 30–35 (first principle calculations, Raman measurements combined with Liddane-Sachs-Teller equation)^50–52^. The values experimentally reported for thin films can go down to 15–20 (see ref.^11^. and the present study). There are some theoretical studies suggesting a hardening of the soft modes in epitaxial thin films^53^. This can potentially lead to lower values of the dielectric constant along the direction perpendicular to the electrodes due to the strain constrains imposed by the substrate, especially in the case of ultra-thin films. Other theoretical studies suggest that it is possible to reach values as low as 10 in thin films^54^. One can conclude that our experimental results, based on the “static” C-V measurement, are in agreement with previous reports in literature regarding the value of the static dielectric constant in very low thickness thin films.

In order to further test the results presented until now, we have calculated the static dielectric constant using density functional perturbation theory calculations implemented in the Quantum Espresso simulation package^55^. The method for calculating the static dielectric constant has been developed by Gonze *et al*.^56^ and has been used successfully to calculate the dielectric function for hybrid organic/inorganic halide perovskites^57^. For simplicity’s sake, we have used PbTiO_3_ as a test material in three scenarios: completely relaxed bulk, strained bulk and thin film. The obtained values are approximately 29 in the case of a bulk unstrained crystal, 32.35 for the strained bulk and about 32.3 for a 5 unit cells film with SRO contacts with the same basal strain as in the previous bulk case. These results are comparable to the experimental values and confirm that the background dielectric constant in PZT crystals or epitaxial films is very low. The details of the calculations can be found in the SI.

There are other aspects that have to be discussed:

- The increase of the static background dielectric constant as the thickness increases. This can be explained by the occurrence of structural defects as revealed by the TEM investigations (results presented in Fig. 3b). One can see in the low magnification TEM images that, as the thickness increases, more and more defects occur in the film (dislocations, grain boundaries, domains and domain walls).
- The constant capacitance in the case of “static” C-V compared to the voltage dependent capacitance in the case of the “dynamic” C-V. This behavior can be explained assuming the presence of Schottky type contacts at the electrode interfaces. As already mentioned in the Introduction section, the presence of Schottky type contacts at electrode interfaces in ferroelectric capacitors is already demonstrated and accepted in the literature^16–20,58,59^. Schottky contacts assume the presence of space charge (depletion) regions at the electrode interfaces, with voltage dependent thickness translated into voltage dependent capacitance^60^ as can be seen in “dynamic” C-V characteristics (Fig. 2a). The relative capacitance variation with the applied voltage, after polarization switching (saturated polarization), reduces from about 15% for the 150 nm thick sample to about 5% for the 20 nm thick sample. This result suggests that, as the thickness decreases, the metal-ferroelectric-metal (MFM) structure tends towards full depletion. Therefore, in the case of “dynamic” C-V, the effect of the Schottky contacts will be less visible at small thicknesses and it is expected to disappear in very thin films, these being fully depleted at any voltage except the voltage range where the polarization switching takes place. On the other hand, in the case of “static” C-V, the films are fully depleted at any thickness because the free carriers from the ferroelectric film are blocked at the interfaces to compensate the polarization charges. As a consequence, the capacitance is nearly constant at any voltage outside the range where the polarization switching takes place.
- The frequency dependence of the dielectric constant confirms that this quantity decreases as the thickness of the epitaxial film is decreased (see details in SI). One interesting aspect is that, for the 20 nm thick film the frequency dependence can be fairly well simulated with a simple Debye equation, as shown in the Supplementary Information.

In summary, it was shown that the static background dielectric constant at low frequencies, in ultra-thin epitaxial ferroelectrics, can have low values, of about 10–15, and can be even lower in ideal, defect free, mono-domain ferroelectric layers. The large values reported in the literature are resulting from extrinsic contributions associated to structural defects, mainly ferroelectric domains and domain walls, but also other structural defects, that occur as the films start to relax when the thickness is increased. It was also evidenced that the natural state of an epitaxial ferroelectric in mono-domain state, with no applied *dc* voltage, is that of full depletion. Once step *dc* voltage is applied the state may change to partial depletion in thick films but remains of full depletion in very thin films. These findings can have an important impact in the analysis of conduction mechanisms in ferroelectrics as well in developing models to simulate the characteristics of ferroelectric-based devices.

## Methods

### Samples

The samples were grown by pulse laser deposition (PLD) on single crystal SrTiO_3_ (STO) substrates. The bottom electrode was a 20 nm thick layer of SrRuO_3_ (SRO). Top SRO/Pt electrodes were deposited for electrical measurements, with area of 0.01 mm^2^. The epitaxial quality of the structures was analyzed by transmission electron microscopy (ARM-200F from JEOL). Details about the deposition process and about the structural characterization can be found in previous publications^61,62^.

### Electric measurements

Polarization and current hysteresis loops were recorded using a model TF2000 ferritester from AixACCT. Capacitance and dielectric losses were recorded using a LCR bridge model Hioki 3536 or a HP 4194A impedance analyzer. The voltage pulses were applied using a Keithley 6517 electrometer. All data were recorded using special designed acquisition programs. All the measurements were performed at room temperature.

### Numerical calculations

Density functional perturbation theory calculations were performed using the Quantum Espresso distribution and the included Phonon package.

## Supplementary information

Low value for the static background dielectric constant in epitaxial PZT thin films
