# Supplementary material for: Low value for the static background dielectric constant in epitaxial PZT thin films
Source: Sci Rep. 2019 Oct 11;9:14698. doi: 10.1038/s41598-019-51312-8 (PMC6789001; doi:10.1038/s41598-019-51312-8)
Supplement: Supplementary file 1 — Low value for the static background dielectric constant in epitaxial PZT thin films [file 41598_2019_51312_MOESM1_ESM.pdf]

Low value for the static background dielectric constant in epitaxial PZT thin films

Georgia Andra Boni, Cristina Florentina Chirila, Luminita Hrib, Raluca Negrea, Lucian Dragos Filip, Ioana Pintilie, Lucian Pintilie

National Institute of Materials Physics, Atomistilor 405A, Magurele, Ilfov, Romania

Supplementary Information

Static and dynamic C-V measurements

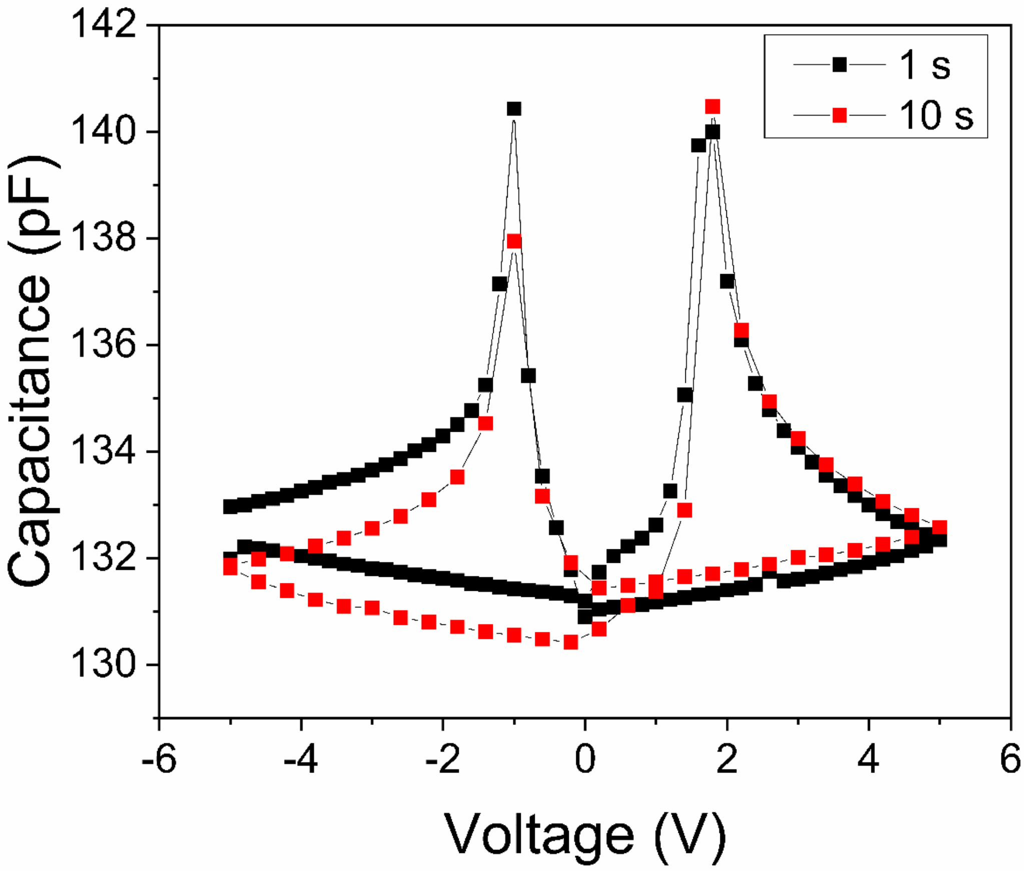

Fig. SI1 C-V characteristics obtained at different waiting times for the MFM capacitor with 150 nm thickness of PZT. The frequency of the *a.c.* voltage was 100 kHz, and amplitude 0.1 V.

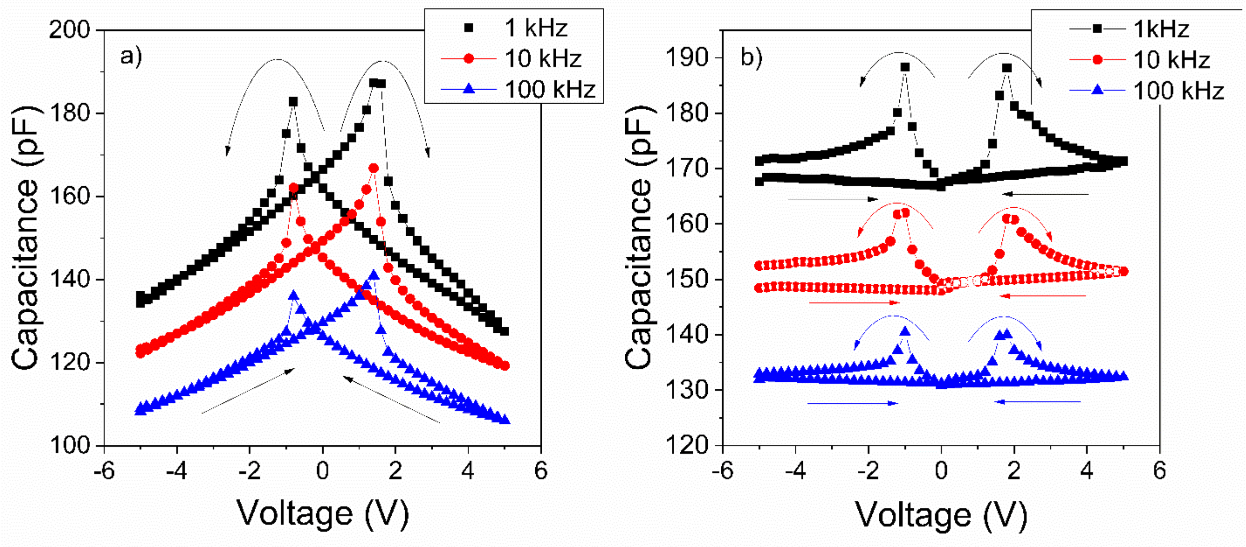

Fig. SI2 C-V characteristics, at different frequencies of the *a.c.* voltage, for the MFM capacitor with 150 nm thick PZT layer. a) dynamic measurement; b) static measurement. The amplitude of the *a.c.* voltage was 0.1 V in all cases.

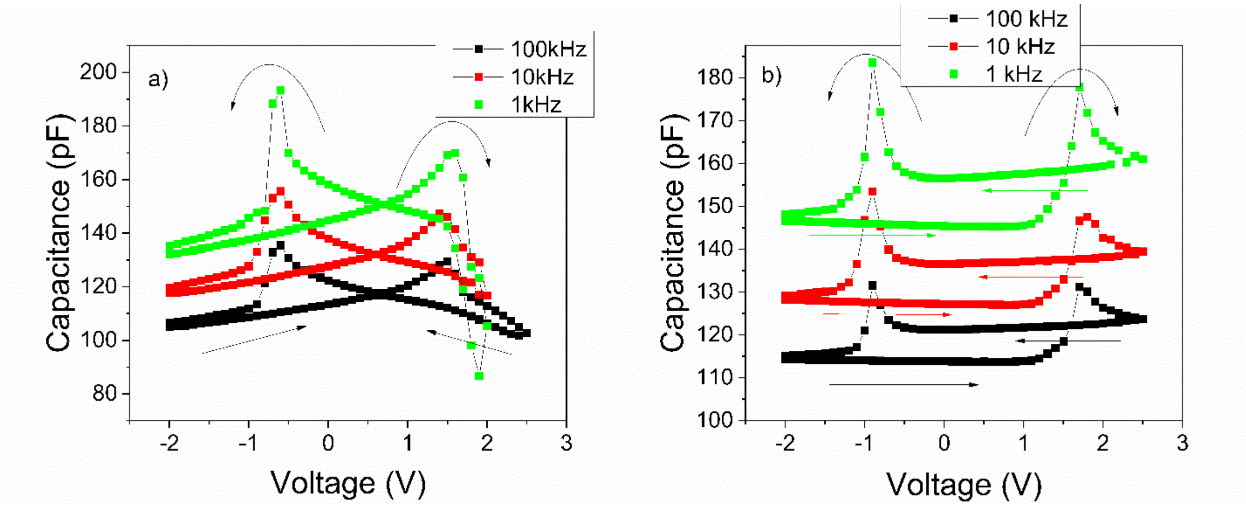

Fig. SI3 C-V characteristics, at different frequencies of the a.c. voltage, for the MFM capacitor with 20 nm thick PZT layer. a) dynamic measurement; b) static measurement. The amplitude of the *a.c.* voltage was 0.1 V in all cases.

Rayleigh analysis

Rayleigh analysis was performed following the procedure proposed by Hall et al. (see Refs 42 and 43 in the main text). Below is an example for the 50 nm thickness sample (Fig. SI4).

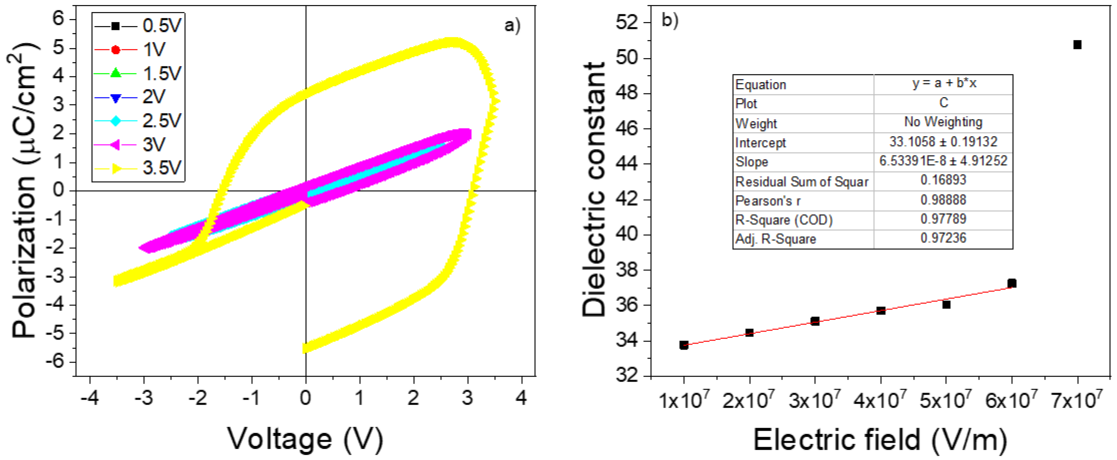

Fig. SI4 a) hysteresis loops recorded at low electric fields (well below the coercive field); b) the Rayleigh representation and the corresponding linear fit.

The linear fit has a confidence of almost 99 %. The intercept gives the dielectric constant at zero electric field, and the obtained value is about 33. The slope gives the Rayleigh constant, and the obtained value is about $6x10^{-8}$ m/V. This is five orders of magnitude lower than in ceramics (see the works of Hall and collaborators, or the work of Eitel et al. in Ref. SR1). This can be explained by the fact that the very thin epitaxial films are mon-domain (see TEM results presented in Figure 3 b) in the main text, as well as the PFM analysis presented in Ref SR2), while ceramic films are poly-domain, with domain walls very sensitive to any applied electric field. In any case, the dielectric constant at zero field is in very good agreement with the value extracted from our C-V measurements. The dielectric constants obtained for the other two samples, applying the same procedure, are about 24 (20 nm) and 63 (150 nm), with Rayleigh constants of about $7x10^{-8}$ m/V (20 nm) and $3x10^{-6}$ m/V (150 nm). The increase of the Rayleigh constant in the thicker films may be related to the presence of some 90^{0} domains, as revealed by TEM investigations (see Figure 3b) in the main text). Comparing these values with those obtained from our C-V measurements, one can observe the followings: for the 20 nm thick sample the values are between 24 (Rayleigh) and

26 (dynamic and static C-V at 0 V), thus if any polarization contribution is present, this is below 10 %; for the 50 nm sample the values are between 33 (Rayleigh) and 40 (dynamic C-V at 0 V), thus the polarization contribution may be around 20 %; for the 150 nm sample the values are between 63 (Rayleigh) and 100 (dynamic and static C-V at 0 V), with a polarization contribution of about 40 %.

Comparing the results of the Rayleigh analysis with those from C-V measurements one can assume that the dielectric constant at zero electric field is more appropriate to be named "static background dielectric constant", while the dielectric constants extracted from C-V measurements are affected by some contribution from polarization, this contribution increasing with thickness as ferroelectric domains develop in the film

Frequency dependence of dielectric constant

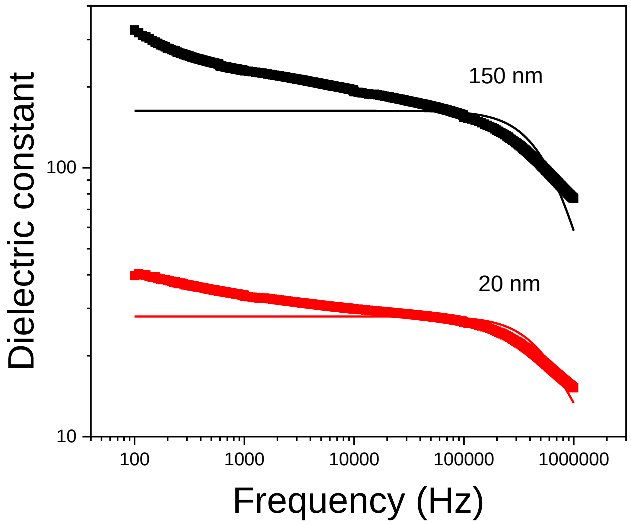

Fig. SM4 The frequency dependence of background dielectric constant for MFM capacitors with 150 nm and 20 nm thick PZT layers. The markers are experimental data; the line are fits using a simple Debye relaxation equation.

The equation for the real part of the relative dielectric constant is:

$$\varepsilon(\omega) = \varepsilon_\infty + \frac{\varepsilon(0)-\varepsilon_\infty}{1+\omega^2\tau^2} \qquad \text{(ESM1)}$$

Here, ε_∞ is the relative dielectric constant in the optical domain, $\varepsilon(0)$ is the relative static dielectric constant, ω is the pulsation of the *a.c.* voltage and τ is the time constant of the capacitor. The values

used for the fits in Fig. SM4 are: $\varepsilon_\infty = 6$, $\varepsilon(0) = 163$ and $\tau = 0.2$ μs for thickness of 150 nm; $\varepsilon_\infty = 6$, $\varepsilon(0) = 28$ and $\tau = 0.2$ μs for thickness of 20 nm. One can observe that the trend for ε(0) is to decrease with decreasing the thickness of the ferroelectric layer, as experimentally observed in Fig. 3a) in the main text.

One can observe that the equation ESM1 describes relatively well the experimental frequency dependence for frequencies above 100 kHz but at lower frequencies there is a significant deviation from the experimental data. The deviation can be attributed to the structural defects acting as trapping centers. The trapped charges may respond to the small amplitude *ac* voltage used for capacitance measurements, leading to increased values of the capacitance and thus, of the dielectric constant.[SR3] The deviation is larger for the sample of 150 nm thickness, explainable by the fact that there are more structural defects acting as traps than in the sample of 20 nm thickness. This is in agreement with the results of TEM investigations presented in Fig. 3 b) of the main text.

The equation ESM1 cannot explain why the value of *ε(0)* is decreasing with thickness. However, is showing the same trend as experimental data. An interesting situation is when $\omega^2\tau^2 << 1$, which can happen when the frequency of the *a.c.* signal approaches zero or when τ is very small. A small value for τ implies either a small capacitance, or a small resistance. Assuming that the capacitance is always finite then τ approaches zero if the resistance of the film is close to zero. This may happen in ultra-thin ferroelectric films showing increasing density of free carriers with decreasing the thickness.[SR2]

Numerical calculations:

As mentioned in the main manuscript we have performed DFPT calculations in order to obtain the static dielectric constant of $PbTiO_3$. It has a simple tetragonal unit cell containing only 5 atoms which allows for very small calculation times while retaining the physical properties of interest. We have studied three cases:

a) the completely relaxed bulk.

b) strained bulk

c) strained thin film between two $SrRuO_3$ electrodes.

We have started from the experimental values for the atomic coordinates and unit cell sizes found in Ref. SR4. The kinetic energy cutoff was 100 Ry and the Brillouin zone was sampled in a 5x5x5 Monkhorst-Pack uniform k-point grid for all calculations except for the thin film case where a grid

of 5x5x1 was used instead. For the case a) the structure was completely relaxed on all axes and atomic coordinates while for case b) and c) only the z direction was allowed to relax. This was due to the fact that cases b) and c) are supposed to simulate an epitaxial growth of $PbTiO_3$ forced to adapt to the $SrTiO_3$ substrate.

After the structural relaxation was performed, phonon calculations were started at Gamma symmetry point in order to obtain the static dielectric constant tensor. The values obtained for the static dielectric constant along the polar axis z for all three cases are summarized in the following

Table.

	Unstrained bulk	Strained bulk	Strained thin film
ε(0)	28.57	32.35	32.27

Bibliography

SR1 Eitel R. E., Shrout T. R., & C. A. Randall, Nonlinear contributions to the dielectric permittivity and converse piezoelectric coefficient in piezoelectric ceramicsJ. Appl. Phys. **99**, 124110 (2006)

SR2 Pintilie, L., Ghica, C., Teodorescu, C. M., Pintilie, I., Chirila, C., Pasuk, I., Trupina, L., Hrib, L., Boni, G. A., Apostol, N., Abramiuc, L., Negrea, R., Stefan S., & Ghica, D., Polarization induced self-doping in epitaxial $Pb(Zr_{0.20}Ti_{0.80})O_3$ thin films, Sci. Rep. **5**, 14974, (2015)

SR3 Vincent, G., Bois, D., & Pinard, P., Conductance and capacitance studies in GaP, Schottky barriers, J. Appl. Phys. **46**, 5173 (1975)

SR4 Glazer, A. M. & Mabud, S. A., Powder profile refinement of lead zirconate titanate at several temperatures. II. Pure PbTiO3, Acta Crystallographica Section B: Structural Crystalography and Crystal Chemistry 34(4) 1065-1070 (1978)
